# Genome-Wide Identification of ATG Gene Family Members in *Fagopyrum tataricum* and Their Expression during Stress Responses

**DOI:** 10.3390/ijms232314845

**Published:** 2022-11-27

**Authors:** Yue Fang, Shuang Wang, Hula Wu, Chenglei Li, Haixia Zhao, Hui Chen, Xiaoli Wang, Qi Wu

**Affiliations:** 1 College of Life Science, Sichuan Agricultural University, No. 46, Xinkang Road, Ya’an 625014, China; 2 State Key Laboratory of Crop Gene Exploration and Utilization in Southwest China, Sichuan Agricultural University, No. 211, Huimin Road, Wenjiang District, Chengdu 611130, China

**Keywords:** Tartary buckwheat, autophagy, *FtATG8s*, abiotic stress, functional validation

## Abstract

Abiotic stresses such as drought and salinity are major environmental factors limiting plant productivity. Autophagy-related genes are extensively involved in plant growth, development, and adverse stress responses, which have not yet been characterized in Tartary buckwheat (*Fagopyrum tataricum*, TB). In this study, we verified that drought stress could induce autophagy in TB roots. Next, 49 *FtATGs* in the whole genome of TB were identified. All *FtATGs* were randomly distributed in 8 known chromosomes, while 11 *FtATGs* were predictably segmental repeats. As the core component of autophagy, there were 8 *FtATG8s* with similar gene structures in TB, while *FtATG8s* showed high expression at the transcription level under drought and salt stresses. The *cis*-acting element analysis identified that all *FtATG8* promoters contain light-responsive and MYB-binding elements. *FtATG8s* showed a cell-wide protein interaction network and strongly correlated with distinct stress-associated transcription factors. Furthermore, overexpression of *FtATG8a* and *FtATG8f* enhanced the antioxidant enzyme activities of TB under adverse stresses. Remarkably, *FtATG8a* and *FtATG8f* may be vital candidates functioning in stress resistance in TB. This study prominently aids in understanding the biological role of *FtATG* genes in TB.

## 1. Introduction

Plants survive in adverse changing environments, which could affect their growth and development [1]. These environmental stimulators consist of biotic stresses, pathogenic infections, aggressive herbivores, and abiotic stresses, including drought, high temperature, cold, lack of nutrients, and excessive salinity in the soil [2]. Plants usually degrade or restore damaged proteins and organelles to adapt to these pressures. Autophagy, as a conservative degradation pathway, plays a central role in eukaryotic stress response.

After autophagy was first discovered and reported in yeast, more autophagy-related genes were excavated, named *ATG1*, *ATG2*, *ATG3*, etc. [3]. Subsequently, 47 ATG family proteins were identified in *Arabidopsis thaliana*, which was named according to their homology with yeast ATG proteins. Compared to the single-copy genes in yeast, some autophagy genes in plants have multiple copies that form subgroups such as ATG8 and ATG12. As a conserved protein degradation and recycling mechanism in plants, autophagy is a fundamental part of the biochemical and physiological processes of plants, including chloroplast renewal [4], pollen development [5], and seed germination [6]. In parallel to growth and development, autophagy widely responds to abiotic stresses in plants. Ion stress caused by drought and salt stress induces oxidative damage in plant cells, which can be repaired by autophagy under the activation of various biological factors [7]. Silencing *OsATG2* and *OsATG7* inhibits autophagy and reduces wheat salt tolerance [8]. Overexpression of *MdATG18a* enhanced the adaptable ability of apples to drought stress [9].

Among *ATGs*, *ATG8s* are critical players in autophagy. Especially when monitoring autophagy is possible, *ATG8s* become the most remarkable discovery in identifying the *ATG* family. The deletion of *ATG8s* led to the failure of smaller autophagosomes to fuse with lysosomes [10,11]. In addition, *ATG8* also plays an essential role in the adaptation to abiotic stresses in plants. *Arabidopsis thaliana* overproducing *MaATG8f* had better drought resistance and sensitivity to ABA than the wild type [12]. Overexpression of *OsATG8c* significantly increased autophagic activity and tolerance to N starvation. In addition, the salt and drought resistance were enhanced in apples overexpressing *MdATG8i* [13,14]. Altogether, it emphasizes that ATG8s have a crucial effect on improving the Resistance to abiotic stresses of plants. Tartary buckwheat (*Fagopyrum tataricum*, TB) is a grain crop used in food and medicine which attracts attention because of its rich flavonoid content [15]. Although TB with enriched nutrition has strong environmental adaptability to grow in high-altitude barren areas, drought is still the main factor inhibiting TB yield. It is necessary to reveal its stress resistance mechanism and identify the stress resistance-associated gene family. Abiotic stress often leads to the appearance of damaged organelles and proteins. ATG-involved autophagy is a conservative recycling mechanism for plants and an important mechanism for responding to abiotic stress.

However, ATG in TB has not been systematically well established yet. In this study, a comprehensive investigation was performed for *FtATG* family genes in the TB genome. A series of studies on the molecular characteristics and functional verification of *FtATGs*, proposed that *FtATGs* were potentially involved in stress tolerance in TB, laying a foundation for the functional characterization of *FtATGs* in TB.

## 2. Results

### 2.1. Autophagy Occurs in TB under Drought Stress

**Figure 1:**
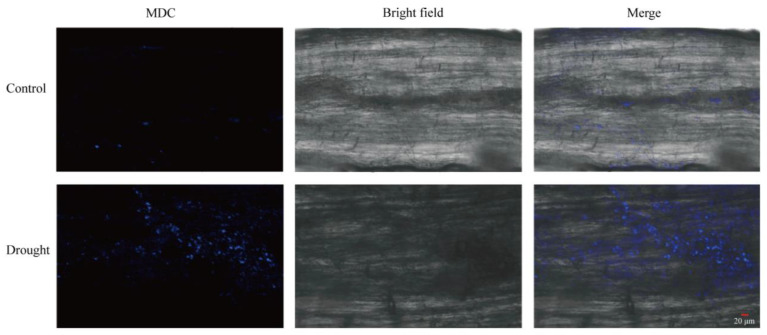
Accumulated autophagosomes in TB roots under drought stress. Autophagosomes were stained by MDC dye. Control indicates TB roots without drought treatment. Scale bars: 20 μm.

Although TB has good stress resistance, drought is still the most severe abiotic stress reducing its yield and quality. To test whether autophagy was involved in drought stress in TB, we visualized the autophagosome abundance in TB roots under drought stress. MDC (Monodansyl-cadaverine) was used for autophagosome staining. Compared to the control group without drought treatment, there was a significant increase of blue fluorescent spots after 24 h under natural drought, indicating autophagosomes accumulated in TB roots (Figure 1). It suggested that autophagy was significantly induced by drought stress in TB.

### 2.2. Genome-Wide Identification of FtATGs in TB

To identify the autophagy-associated genes in TB, 39 *OsATGs*, 29 *NtATGs*, and 47 *AtATGs* were taken as templates to align with genes from the TB gene database using BLASTp (Basic Local Alignment Search Tool for Proteins). Forty-nine presumptive *FtATGs* from 22 subfamilies were identified. The number of genes varied widely among subfamilies. Most genes clustered in the FtATG18 subfamily (9 members) while following in the FtATG8 subfamily (8 members). Some subfamily members were named based on the similarity to known proteins, while some were randomly named (Table S1). The *FtATG* sequences exhibited great differences, and their ORF lengths range from 289 bp (*FtATG12a*) to 7563 bp (*FtTOR*). Additionally, the physicochemical properties of *FtATG* genes and their encoding proteins were also different, which also addressed that *FtATGs* function in a diverse way.

**Figure 2:**
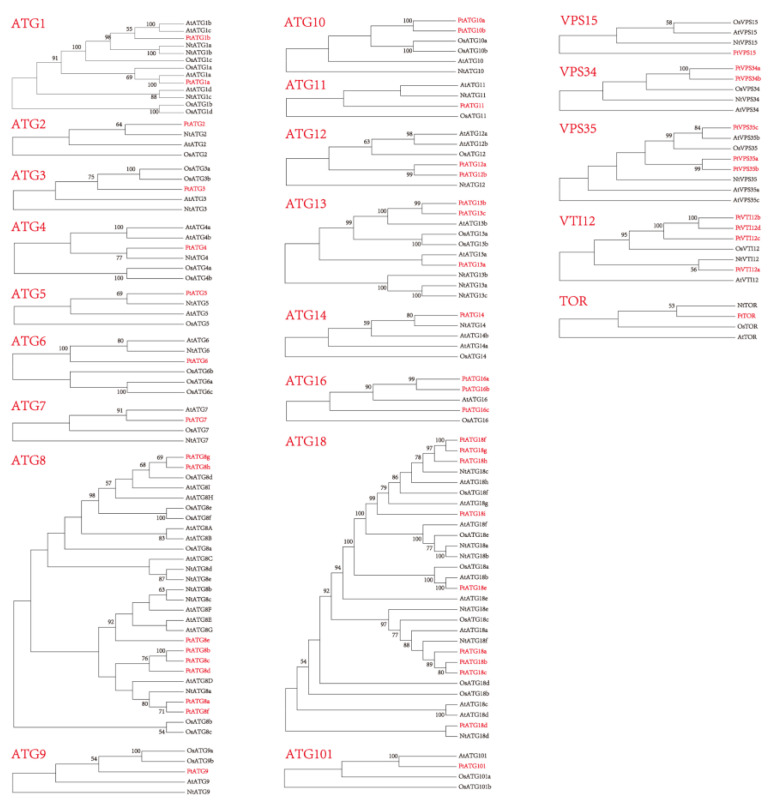
Phylogenetic analysis of ATG proteins from *Fagopyrum tataricum* and other plants. Neighbor-joining trees are constructed with 49 FtATGs of *Fagopyrum tataricum* (Ft), 39 OsATGs of *Oryza sativa* (Os), 29 NtATGs of *Nicotiana tabacum* (Nt), and 47 AtATGs of *Arabidopsis thaliana* (At) using the MEGAX software, with 2000 bootstrap replicates. The proteins of TB are marked in red. The phylogenetic tree shows bootstrap values above 50%.

To explore the phylogenetic relationship among ATGs, 22 Neighbor-joining trees of subfamilies were constructed with 49 FtATGs, 39 OsATGs, 29 NtATGs, and 47 AtATGs (Figure 2). For single-membered subfamilies, FtATGs were often clustered into the same branch as the NtATGs from tobacco. In contrast, for subfamilies with multiple members, FtATGs were usually clustered into the same branch, including ATG10, ATG12, ATG16, VPS34, and VTI12, or clustered into several branches, such as ATG8, ATG18, and VPS35. These results suggested potential functional differentiation of FtATGs in multiple subfamilies.

### 2.3. Phylogenetic Tree Construction and Conserved Motifs of FtATGs

**Figure 3:**
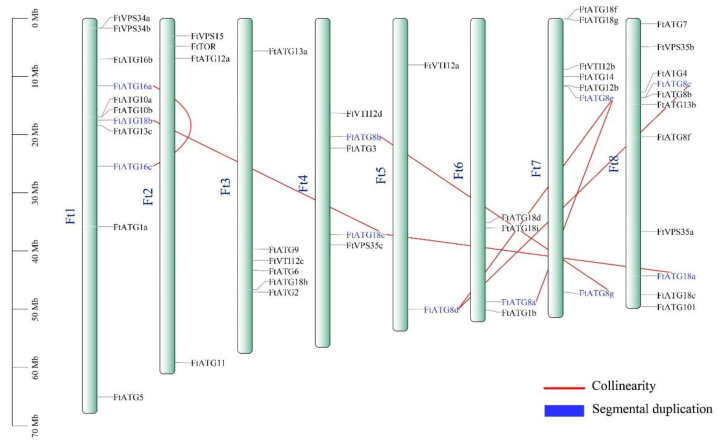
Chromosomal distribution and gene duplication of *FtATGs*. *FtATGs* with fragment duplicates were marked in blue, and the red lines represent the collinearity between two genes.

The chromosomal distribution of 49 *FtATGs* is shown in Figure 3. *FtATGs* were unevenly located in the eight chromosomes of TB. Chromosomes 1 and 8 contained the most *FtATGs*, each having 11 *FtATGs*. In addition, chromosome 7 contained seven *FtATGs*, while chromosome 3 contained six *FtATGs*. The rest of the genes were sparsely located in the remaining chromosomes. Gene replication analysis confirmed that 11 *FtATGs* existed in highly replicated fragments, which constructed seven pairs of collinear genes. The Ka/Ks values of these collinear genes were lower than one, indicating that purification selection was a central impactor for the *ATG* subfamily members.

**Figure 4:**
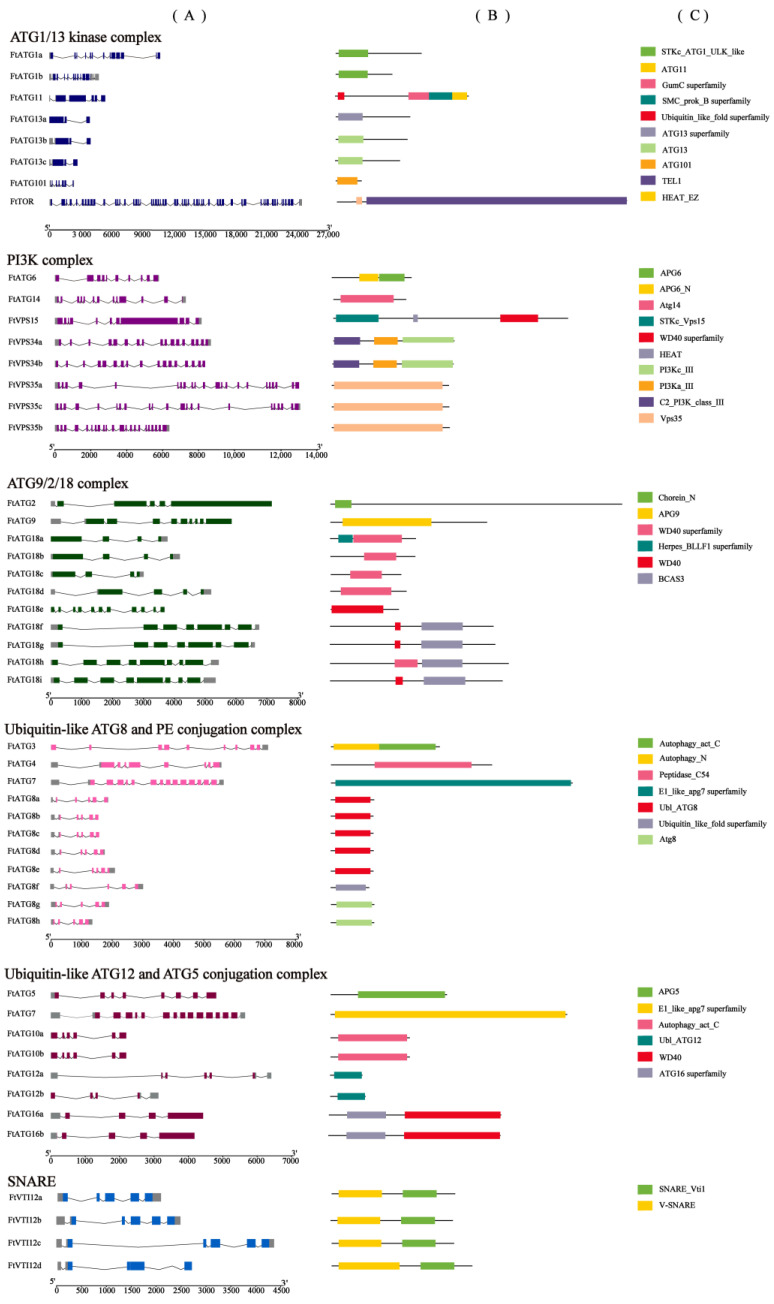
Domains and gene structures of *FtATGs*. (**A**) The exon-intron structures. The colorful rectangles represent exons, except the gray rectangles indicate the untranslated regions (UTRs). Introns are indicated by black lines. (**B**,**C**) Domains of the corresponding genes. The 49 *FtATG* genes were described based on their functions in autophagy.

Next, according to the TB genome annotation and CDD-search online tool, we analyzed specific exons, introns, and conserved domains of *FtATGs* (Figure 4). Based on their functions, FtATGs were categorized into ubiquitin-like ATG12 and ATG5 conjugation complex, SNARE, ATG9/2/18 complex, ubiquitin-like ATG8, and PE conjugation complex, PI3K complex, and ATG1/13 kinase complex. There were significant differences in the number of exons and introns among the subgroups. *FtATG13a* only has three exons, while *FtTOR* has 57 exons. The exon-intron information within the same subgroups showed high similarity. For example, the genes in the *FtATG8* subgroup all have five exons. According to the genome annotation information, *FtATG13a* and *FtATG12b* lacked the non-coding regions at the 5′ end, whereas the non-coding regions at the 3′ end were absent in *FtATG2*, *FtATG9*, *FtVPS34b*, *FtVPS5a*, *FtATG8b*, *FtATG8c*, *FtATG10a*, *FtATG10b*, *FtATG16a*, *FtATG16b*, and *FtVTI12d*, respectively. These results indicated that the genome annotation information might be partially incorrect, which means further improvement is required.

### 2.4. Analysis of FtATGs Expression Pattern under Stress

**Figure 5:**
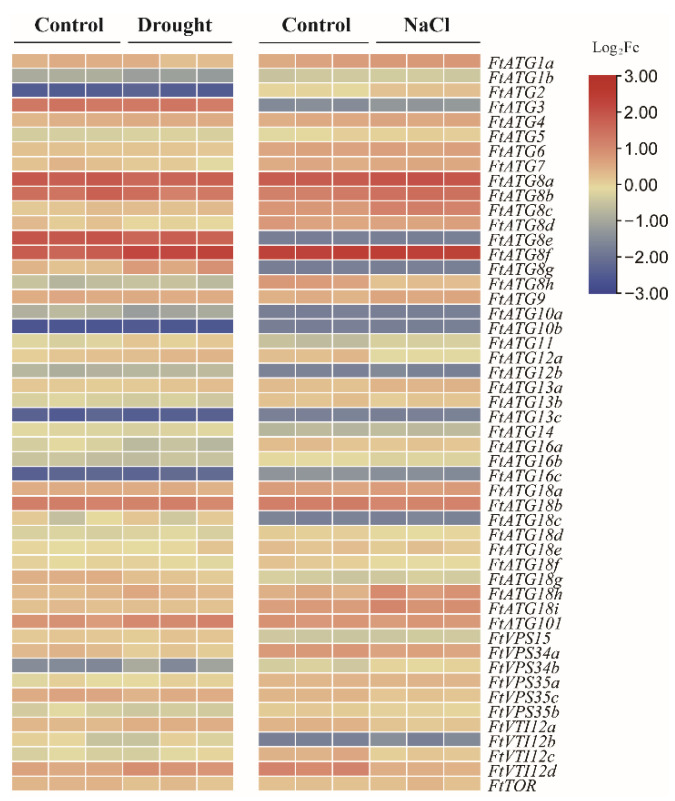
The expression heatmap of *FtATGs* under drought and salt stress. Each row represents one gene, and every three columns represent different replicates for each treatment.

To identify the core candidates for *FtATGs* in response to drought and salt stress, we analyzed their expression pattern according to the transcriptome data of TB under the corresponding stress (Figure 5; Table S2). The results showed that *FtATGs* exhibited different expression patterns. Some genes expressed at extremely low levels, such as *FtATG10b*, *FtATG13c*, and *FtATG16c*. Among the rest of the *FtATGs*, the members of the *FtATG8* subfamily were the most prominent. *FtATG8a* and *FtATG8f* showed remarkably high expression, suggesting that they may be potentially important stress resistance genes. Therefore, given the high total number of 49 *FtATGs* identified and the importance of *FtATG8s* in autophagy, we chose to conduct an in-depth study of *FtATG8s*.

### 2.5. Cis-Acting Elements in FtATG8s Promoter

**Figure 6:**
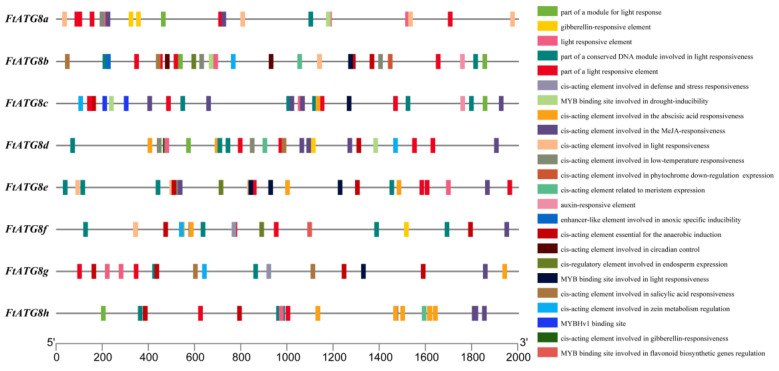
*Cis*-acting elements in the promoter regions of *FtATG8s*. *Cis*-acting elements with analogous functions were presented in the same color as indicated. The colorful rectangles on the right column represent *cis*-acting elements with different functions.

To accurately predict the function of *FtATG8s* and understand the potential regulation mechanism, the *cis*-acting elements in the promoter region of *FtATG8s* were analyzed (Figure 6; Table S3). The results showed that all *FtATG8s* contained anaerobic induction, light, and hormone (methyl jasmonate [MeJA] and abscisic acid [ABA]) *cis*-elements. Among them, the *FtATG8a* promoter had up to 15 light response elements. Four *FtATG8s* (*FtATG8a*, *FtATG8b*, *FtATG8d*, and *FtATG8e*) contain low-temperature response elements. Three *FtATG8s* (*FtATG8a*, *FtATG8f*, and *FtATG8g*) were with defense and stress elements, and only *FtATG8b* had circadian rhythm response elements. In addition, all *FtATG8s* had at least one MYB binding element. *FtATG8f* and *FtATG8g* have MYB binding sites involved regulating flavonoid biosynthesis genes, and *FtATG8a*, *FtATG8b*, and *FtATG8c* have MYB binding sites involved in drought induction.

### 2.6. Prediction of FtATGs Interacting Proteins

**Figure 7:**
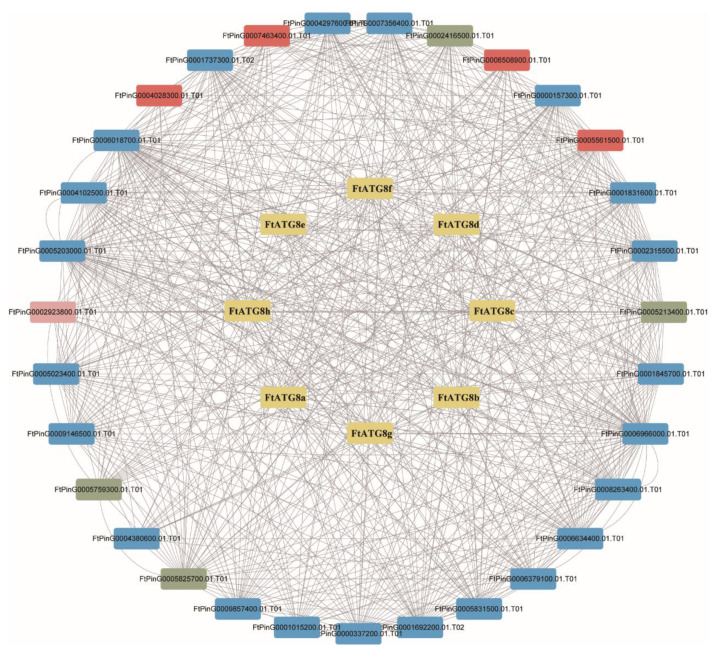
Analysis of the functional interaction network of FtATG8 proteins. FtATG8s were highlighted in yellow. Proteins in red were predicted to encode ubiquitin-related proteins, and the pink indicated a peroxisomal protein. Other FtATGs were shown in blue, and undermined proteins were in green.

For the proteins interacting with FtATG8s in TB, an interaction network involving FtATG8s was established (Figure 7). Results showed that FtATG8s were more likely to interact with other members of the ATG family and possibly with four ubiquitin-related proteins such as FtPinG0004028300.01.T01, indicating that autophagy can also influence the protein degradation in the ubiquitous pathway. Besides, FtATG8s can interact with a peroxisomal biogenesis protein FtPinG0002923800.01.T01, suggesting autophagy might recruit peroxisome biogenesis in TB.

### 2.7. Correlation Analysis between FtATG8s and Stress Resistance-Related Transcription Factors

**Figure 8:**
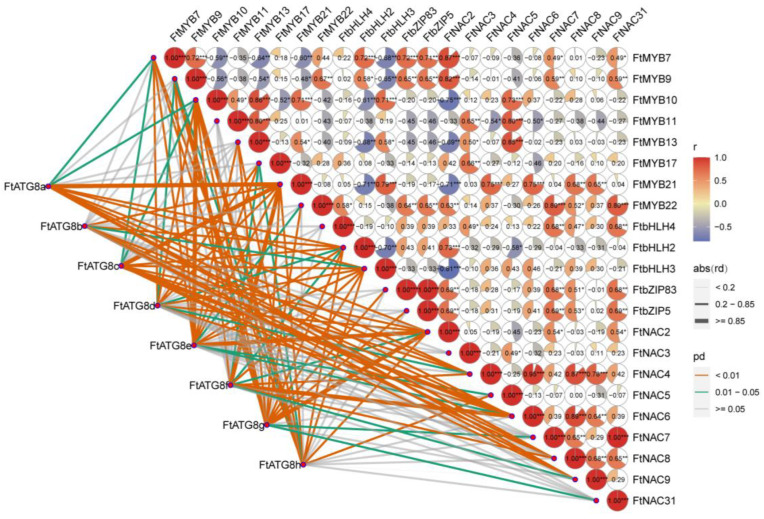
Correlations of expression patterns between *FtATGs* and other transcription factors. RStudio was applied for this visualization. Line thickness mapping absolute value of correlation, color mapping p value credibility (* *p* < 0.05, ** *p* < 0.01, *** *p* < 0.001). *FtMYB7* (FtPinG0003734600.01), *FtMYB9* (*FtPinG0002001900.01*), *FtMYB10* (*FtPinG0002706600.01*), *FtMYB11 (FtPinG0008533900.01), FtMYB13* (*FtPinG0005410000.01*), *FtMYB17* (*FtPinG0006925500.01*), *FtMYB21* (*FtPinG0004929500.01*), *FtMYB22* (*FtPinG0003119800.01*), *FtNAC2* (*FtPinG0005692100.01*), *FtNAC3* (*FtPinG0000381200.01*), *FtNAC4* (*FtPinG0005791100.01*), *FtNAC5* (*FtPinG0006190400.01*), *FtNAC6* (*FtPinG0005624400.01*), *FtNAC7* (*FtPinG0005167000.01*), *FtNAC8* (*FtPinG0002252000.01*), *FtNAC9* (*FtPinG0002967400.01*), *FtNAC31* (*FtPinG0005167000.01*), *FtbHLH2* (GenBank:*KU296218*), *FtbHLH4* (*FtPinG0002267300.01*), *FtbHLH3* (GenBank:*KU296217.1*), *FtbZIP83* (*FtPinG0002143600.01*), *FtbZIP5* (*FtPinG0003196200.01*).

Here, to explore the potential transcriptional regulation mechanism of *FtATG8s*, the correlation analysis between the expression of *FtATG8s* and related transcription factor genes from TB under drought and salt stress was performed (Figure 8). The results implied that *FtATG8a*, *FtATG8c*, and *FtATG8g* exhibited a high correlation with numerous stress-related transcription factors in TB, suggesting that *FtATG8s* may be extensively transcriptionally regulated. These findings also revealed a high degree of correlation between the expression levels of *FtATG8s* and most stress-related transcription factors such as *FtMYB10*, *FtMYB21*, *FtbHLH2*, *FtNAC2*, and *FtNAC6*, which further indicates that *FtATG8s* may be involved in the salt and drought stress in TB.

### 2.8. Expression Analysis of FtATG8s in Different TB Tissues

**Figure 9:**
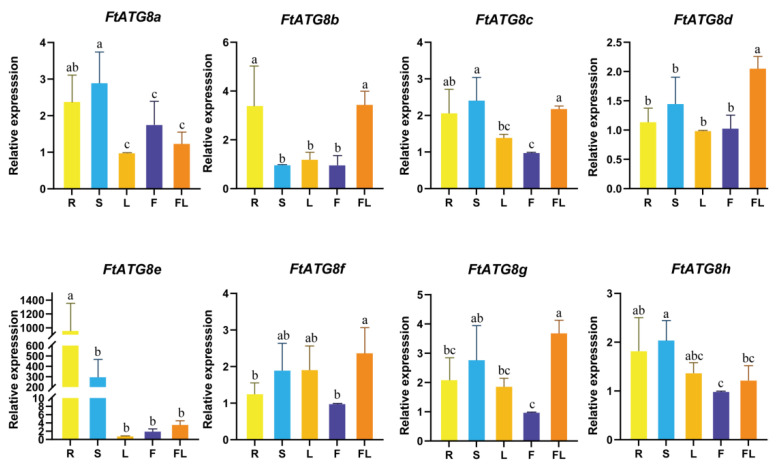
qRT-PCR analysis of FtATG8s in different tissues. R (root), S (stem), L (leaf), F (flower), FL (seed). The minimum expression levels of each gene in the five tissues were set to 1. Error bars represent SD calculated from three experiment repeats. Letters on the bars represent significant differences among (α = 0.05, Duncan) tissues.

The *FtATG8s* in TB may be related to the spatiotemporal specificity. Thus, we investigate the tissue-specific expressed *FtATG8s*. Expression patterns of such genes are diverse in the tissues of TB, showing that *FtATG8s* have different functions in TB (Figure 9). *FtATG8b* was significantly expressed at high levels in roots and seeds. In addition, the expression of *FtATG8a* and *FtATG8e* was much higher in roots and stems compared to other tissues. Meanwhile, *FtATG8d*, *FtATG8f*, and *FtATG8g* had the highest expression in seeds.

### 2.9. Expression Modes of FtATG8s under Abiotic Stresses

**Figure 10:**
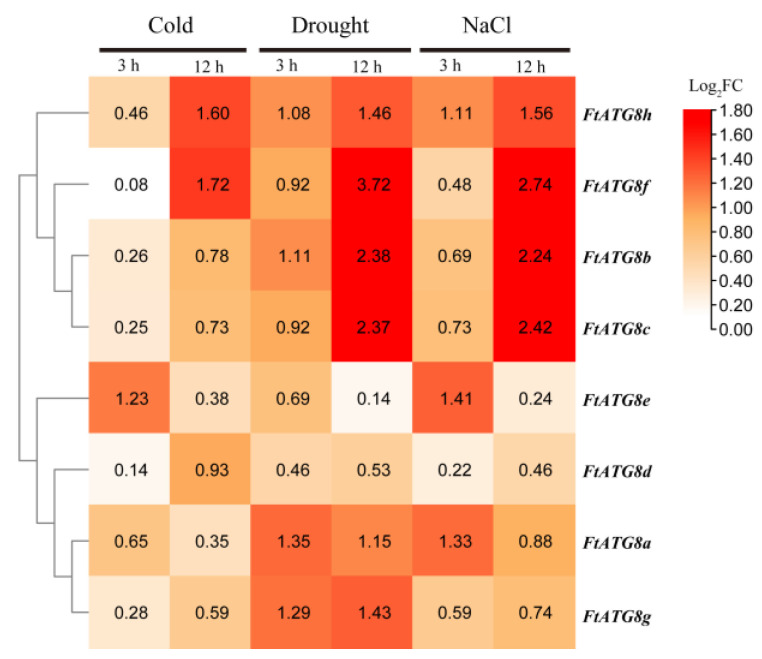
Relative expression of *FtATG8* genes under cold, NaCl, and drought treatments. The expression of *FtATG8s* was set to “1” after 3 and 12 h without stress. The R package was used to generate a heat map based on the mean log_2_FC values of three biological replicates.

Since TB often grows in high-altitude barren areas, cold is the major abiotic stress it faces, except drought. Therefore, we studied the expression patterns of *FtATG8s* under cold, drought, and salt stress. As shown in Figure 10, most *FtATG8s* are up-regulated at least at one time point under different stresses. Remarkably, *FtATG8s* show significantly different expression patterns. The expression of *FtATG8a* and *FtATG8e* peaked at 3 h, while the rest of *FtATG8s* peaked at 12 h, especially *FtATG8f*. These results further illustrated the spatiotemporal specificity of *FtATG8s* in response to abiotic stress. *FtATG8a* and *FtATG8f* are significantly up-regulated under stress at the first and second time points, respectively.

### 2.10. Overexpression of FtATG8a and FtATG8f May Enhance Drought Resistance in TB

**Figure 11:**
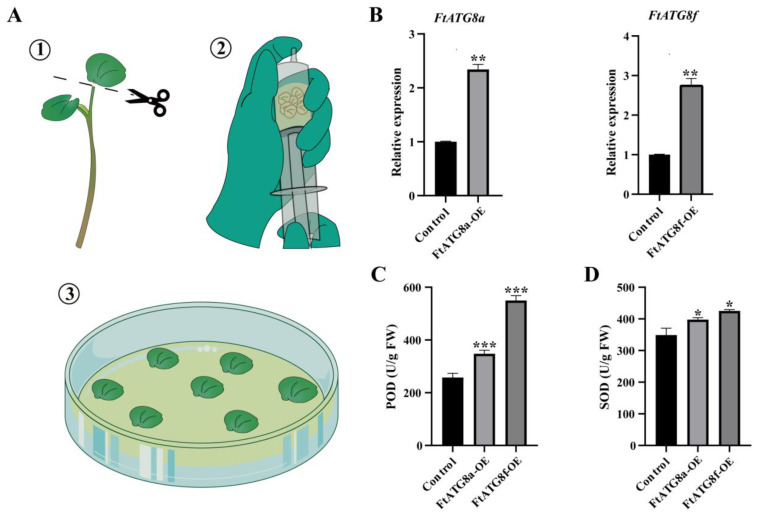
Effects of transient overexpression of *FtATG8a* and *FtATG8f* on antioxidant enzyme activity in TB. (**A**) Transient transformation of TB cotyledon by pCHF3-*FtATG8a*-YFP (termed *FtATG8a*-OE), pCHF3-*FtATG8f*-YFP (termed *FtATG8a*-OE), and pCHF3-YFP (termed Control) mediated by *Agrobacterium* tumefaciens in vacuum infiltration. (**B**) Relative expression of *FtATG8a* and *FtATG8f* after transient expression. (**C**,**D**) Determination of POD and SOD under abiotic stress. Error bars indicate the SD of three independent experiments. Asterisks indicate significant differences (* *p* < 0.05, ** *p* < 0.01, *** *p* < 0.001).

To explore the impacts of overexpression of *FtATG8a* and *FtATG8f* on drought stress, 2-week-old TB cotyledons were transformed by vacuum infiltration under the same growth conditions (Figure 11A). Compared to the control group, after transient transformation of *FtATG8a* and *FtATG8f* for 16 h, the expression level of *FtATG8a* increased to 2.34 times, and the expression level of *FtATG8f* was 2.74 times (*p* < 0.01) (Figure 11B). The determination of antioxidant enzyme activity after 12 h of drought stress suggests that the SOD and POD contents of the experimental group are significantly higher than those in the control group (Figure 11C,D). This suggests that overexpression of *FtATG8a* and *FtATG8f* strengthens the drought tolerance in TB, which might be related to the regulation of ROS levels.

## 3. Discussion

Drought, salt, and climate stress are the main natural environmental factors affecting the physical spread of plants, limiting agricultural plant productivity and threatening food security [16]. How plants adapt to these unfavorable environmental factors is a fundamental biological problem. In addition, enhancing plant resilience is vital to agricultural fertility and stability, as poorly resilient crops consume excessive water and fertilizer [17]. Autophagy extensively functions in plant growth, development, and stress response [18]. The in-depth study of autophagy in TB contributes to understanding the molecular mechanism of plant response to abiotic stress and promotes the breeding of excellent stress-resistant varieties. Besides *Arabidopsis*, autophagy genes are also found in higher plants, such as 32 *OsATGs* in rice [19], 26 *CsATGs* in Sweet Orange [20], 80 *CsATGs* in tea [21], etc. We screen 49 *FtATGs* genes in the TB genome. Similar to Arabidopsis and other plants, we found that FtATG8 and FtATG18 contained multiple members. However, TB had three *FtATG16s* and four *FtVTI12s*, respectively. It was different from Arabidopsis, with only one *AtATG16* and *ATVTI12*. This result suggested that the autophagy genes in TB were species-specific.

The phylogenetic tree analysis showed that among *Arabidopsis*, *Nicotiana tabacum*, and *Oryza sativa*, *FtATGs* had the highest homology with *Nicotiana tabacum*, which is also a dicotyledon. Notably, FtATG8 and FtATG18 are the two most abundant subgroups, and their members share similar conservative domains and high-frequency gene duplication events. These results suggest that *FtATG8s* and *FtATG18s* may have functional redundancy, assuming their expression patterns may have space-time specificity. Our results also deepen the possibility of this speculation. Several duplication events were predicted in the *FtATG* family. Evolutionary events such as duplication can extend the gene family members, while some point mutations in exon regions, as well as the regulatory site, could affect the expression and function of duplicated members [22,23]. It seems that *FtATG* family genes have extended under evolutionary pressures, and some new members have acquired new functions.

Analysis of *cis*-acting elements in the gene promoter regions allowed the prediction of potential mechanisms of gene regulation [24]. The results showed that phytohormone-related acting elements were present in all *FtATGs*, indicating that *FtATGs* played an important role in TB growth. Different environmental stimulatory elements were found in the promoter regions of some *FtATGs*, suggesting that *FtATGs* crucially functioned in response to stress. The presence of many light-responsive elements suggested that light was an important signal regulating *FtATGs* expression.

*ATG8* genes are critical in the plant abiotic stress response. We revealed that all *FtATG8s* were inductively expressed under drought, low temperature, or salt stress and showed different expression patterns. Among them, *FtATG8a* was upregulated under drought conditions while *FtATG8e* was significantly downregulated, similar to *CsATGs* in sweet orange [25], *CaATGs* in pepper [26], etc. The reason may be due to different abiotic stresses regulating autophagy through different pathways [27]. Moreover, autophagy is a long-term regulatory process. In grapes, genes such as *Vvatg1b* are induced in 20 days of drought stress but not in 24–48 h [28]. Some autophagy genes respond at the early stage, and some respond later, which is also supported by our results.

Due to its species specificity, the transient transformation of TB is challenging, difficult to manipulate, and has a low positivity rate [29]. Compared with general syringe injection methods, vacuum infiltration is simple to operate and has better applicability among tissues. This technique has been successfully applied in *Arabidopsis* [30], *Brassica oleracea* [31], and *Prunus armeniaca* [32]. In this experiment, the TB cotyledons were successfully transformed by *Agrobacterium*-mediated vacuum infiltration. It validated that their overexpression of *FtATG8a* and *FtATG8f* could enhance resistance to drought stress in TB.

Since the mechanism of autophagy has been fully studied, it is necessary to unravel its possible regulatory relationship. AtTGA9, a positive regulatory transcription factor for autophagy, was identified in *Arabidopsis* [33]. The promoter element analysis illustrated that *FtATG8* promoters have MYB binding elements, and *FtATG8e* has three MYB binding elements. Combined with the high correlation of MYB transcription factors in the correlation analysis between *FtATG8s* and abiotic stress-related transcription factors of TB, it indicates that MYB transcription factors may regulate *FtATG8s*. Moreover, plants often adapt to the environment by synthesizing anthocyanins and other flavonoids upon abiotic stress, which also relates to autophagy. It reported that the deficient ATG in *Arabidopsis* caused the low vacuolar content of anthocyanin [34]. In this study, we also observed that *FtATG8f* and *FtATG8g* might regulate genes functioning in flavonoid biosynthesis together with MYB. This result strongly proposed that *FtATG8s* responding to stress in TB may not only be limited to the elimination of peroxidase but also be involved in the metabolism of secondary plant products.

## 4. Materials and Methods

### 4.1. Identification of FtATG Genes

In order to screen *ATG* genes in TB, we downloaded the genome data from the website http://www.mbkbase.org/Pinku1/, accessed on 12 April 2022. *Arabidopsis thaliana*, *Nicotiana tabacum*, and *Oryza sativa* ATG proteins from the website (https://www.ncbi.nlm.nih.gov/, accessed on 25 April 2022) were used as queries to search for TB proteins using BLASTP. All presumed *ATG* genes were submitted to the Pfam database (http://Pfam.xfam.org/, accessed on 25 April 2022) to verify the existence of ATG domains. The *ATG* genes of all identified in TB were named *FtATGs.*

### 4.2. Autophagosome Monitoring of TB Roots

2-week-old hydroponic TB seedlings were subjected to natural drought stress by removing water from the culture pots for 24 h and incubated with 300 μM Monodansyl-cadaverine (Solarbio, Beijing, China) dye for 15 min under vacuum. Subsequently, after the remaining Monodansyl-cadaverine dye was washed with PBS, roots were observed under a laser scanning confocal microscope (excitation light wavelength was 488 nm).

### 4.3. Analysis of Main Characteristics of ATG Family Members in TB

The phylogenetic tree of ATG protein sequences of TB and three other species (*Arabidopsis thaliana*, *Nicotiana tabacum*, and *Oryza sativa*) was constructed using the NJ method in MEGA 10 with 2000 bootstrap replicates, which was visualized by iTOL (https://iTOL.embl.de/, accessed on 6 May 2022). We analyzed all *FtATG* distributions, gene structures, motifs, and *cis*-acting elements, using TB genome annotation information. Then, we visualized the results by using TBtools [29]. The characteristic motifs of *FtATGs* were defined by MEME (http://MEME-suite.Org/tools/meme, accessed on 8 May 2022). Furthermore, in order to analyze the *cis*-acting function in the promoter region of the *FtATGs*, we truncated the 2 kb sequence upstream of the start codon of the *FtATG* genes, which was recorded in the PlantCARE Database (http://bioinformatics.psb.ugent.be/webtools/plantcare/html/, accessed on 9 May 2022). The gene duplication events and collinearity analysis of *FtATGs* were evaluated and performed.

### 4.4. Heat Map of the FtATG Expression Patterns under Stresses

The heat map was created to show the *FtATG* expression patterns under stress treatments using TBtools with available transcriptomic data (Table S2). Transcriptome data under salt stress were obtained from the NCBI with accession number PRJNA528524 (http://www.ncbi.nlm.nih.gov/Traces/sra/, accessed on 22 May 2022). Transcriptome data upon drought treatment (20% PEG6000) were generated confidentially (unpublished).

### 4.5. Plant Materials and Treatments

In this study, the TB variety “Xiqiao No. 2” was the plant material grown at Sichuan Agricultural University, Ya’an, China. The buckwheat cotyledon grown in hydroponic culture for 14 d were subjected to the following abiotic stress treatments (20% PEG6000 (*w*/*v*) and 150 mM NaCl and 4 °C). The cotyledons were collected at 3 h and 12 h, then immediately frozen in liquid nitrogen for storage at −80 °C refrigerator.

### 4.6. Construction of Protein-Protein Interaction Network

ATG8 protein sequences with string for protein-protein interaction prediction (http://string-db.org/, accessed on 11 June 2022) were combined. Cytoscape (http://www.cytoscape.Org/, accessed on 17 June 2022) was applied to visualize the regulatory network of other proteins and ATG8s.

### 4.7. Correlation Analysis between FtATG8s and Transcription Factors

To predict the function of *FtATG8s*, transcription factors whose functions have been identified from TB, including *MYB* (*FtMYB7*, *FtMYB9*, *FtMYB10*, *FtMYB11*, *FtMYB13*, *FtMYB17*, *FtMYB21*, and *FtMYB22*) *bHLH* (*FtbHLH2*, *FtbHLH3*, and *FtbHLH4*), *NAC* (*FtNAC2*, *FtNAC3*, *FtNAC4*, *FtNAC5*, *FtNAC6*, *FtNAC7*, *FtNAC8*, *FtNAC9*, and *FtNAC31*), *bZIP* (*FtbZIP83* and *FtbZIP5*) were used to analyze. Together with the transcriptome data used in the heat map analysis, the correlation analysis between *FtATG8s* and transcription factors was calculated using the ggcor function in R language 4.2 and the default Pearson correlation coefficient.

### 4.8. Transient Expression of FtATG8a and FtATG8f in TB Cotyledons

To further investigate the function of TB *FtATG* genes, we chose to clone the full coding sequences of *FtATG8a* and *FtATG8f*, respectively. The ORF of *FtATG8a* and *FtATG8f* were PCR-amplified using primers (Table S4). Then, the sequence was inserted into the plant overexpression vector pCHF3-YFP (Vazyme, Nanjing, China). The recombinant plasmid was transformed into TB cotyledons by *Agrobacterium*-mediated vacuum infiltration. Under the same conditions, pCHF3-YFP was instantaneously transformed as a control group. Sixteen hours after transformation, cotyledons from the experimental and control groups were placed on MS plates containing 200 mM mannitol for 16 h; Superoxide peroxidase (POD) and dismutase (SOD) contents were then measured in the experimental and control groups. SOD and POD were measured by the method of Yao [29].

### 4.9. Quantitative Real-Time PCR Analysis

Total RNA was extracted by EASY spin Plant RNA Kit (Aidlab, Beijing, China) as a template for synthesis of the First-strand cDNAs by qPCR (Vazyme, Nanjing, China) using HiScrip^®^ III–RT SuperMix. The qRT-PCR primers (Table S5) were designed with primer 3 (https://www.ncbi.nlm.nih.gov/Tool/primer-blast/, accessed on 29 June 2022). 2xChamQ Universal SYBR qPCR Master Mix (Vazyme, Nanjing, China) was used for qRT-PCR. The amplification procedure was as follows: 34 cycles of 98 °C for 45 s, then 98 °C for 15 s, and 60 °C for 45 s. The relevant expression data were calculated using the 2^−ΔΔCT^ method, while *FtH3* was used as the internal reference gene. Each group contains three replicates.

### 4.10. Statistical Analysis

The experimental data were processed and visualized by GraphPad Prism (GraphPad Prism 8.0, GraphPad Software, San Diego, CA, USA) and shown in mean ± SD. The significant difference was indicated at the 0.05 and 0.01 levels calculated with ANOVA in IBM SPSS Statistics. (IBM SPSS Statistics 22.0, International Business Machines Corporation, Armonk, NY, USA)

## 5. Conclusions

In this study, we revealed that autophagy in buckwheat responds to drought stress and identified 49 *FtATG* genes from the TB genome. The phylogenetic tree constructed and the motif analysis strongly support the *FtATGs* identification. Analysis of the protein interaction of FtATG8s and its expression patterns with stress-related transcription factors showed that *FtATG8s* might be involved in the expression regulation of stress genes. Lastly, stress experiments reveal that overexpression of *FtATG8a* and *FtATG8f* might improve the resistance of TB to stress by strengthening the activity of antioxidant enzymes.

## Supplementary Materials

The following supporting information can be downloaded at: https://www.mdpi.com/article/10.3390/ijms232314845/s1, Table S1: Physical and chemical properties of *ATG* family. Table S2: Transcriptome data. Table S3: *Cis*-acting elements of *FtATGs* promoter. Table S4: Primers used for construct expression vector of *FtATG8a* and *FtATG8f* for expression of recombinant plasmid in Tartary buckwheat using pCHF-YFP vector. Table S5: Primer sequences of RT-qPCR.
